# Simulation-Based Microfluidic Deformation Mapping for Region-Dependent Apparent Young’s Modulus Estimation of Single Cells

**DOI:** 10.3390/bios16090461

**Published:** 2026-08-25

**Authors:** Minhui Liang, Yilong Zhou, Dawei Ming, Jiawei Lyu, Jianwei Zhong, Han Li, Lin Lin

**Affiliations:** 1 School of Mechanical Engineering, Guangxi University, Nanning 530004, China; minhui@gxu.edu.cn (M.L.); 2301180134@st.gxu.edu.cn (Y.Z.); 2411301040@st.gxu.edu.cn (D.M.); 2511300045@st.gxu.edu.cn (J.L.); 2 School of Microelectronics, South China University of Technology, Guangzhou 511442, China; jwzhong@scut.edu.cn; 3 The People’s Hospital of Guangxi Zhuang Autonomous Region, Guangxi Academy of Medical Sciences, Nanning 530021, China

**Keywords:** single-cell mechanophenotyping, microfluidics, deformability cytometry, Young’s modulus, numerical simulation, hydrodynamic deformation

## Abstract

High-throughput microfluidic deformation assays enable label-free single-cell mechanophenotyping by quantifying how cells deform under controlled hydrodynamic loading. These approaches commonly extract deformation-related observables, such as projected area, axis ratio, and deformation index, and use them as indicators for cellular mechanical properties. However, deformation is not solely determined by stiffness; it is a coupled outcome of cell size, local hydrodynamic stress, and intrinsic mechanical response. Therefore, we present a simulation-based microfluidic framework for estimating region-dependent apparent Young’s modulus (*E*, a quantitative indicator characterizing cellular mechanical stiffness) from diameter–deformation measurements at the single-cell level. A three-region microfluidic channel is designed to impose distinct hydrodynamic loading conditions, while numerical simulations establish quantitative maps linking cell diameter, deformation, and *E*. Based on these results, region-specific nonlinear surface models are constructed to invert experimental diameter–deformation measurements into *E* values. Finally, application to primary T cells and K562 cells demonstrates clear region-dependent differences in *E*, highlighting the influence of local loading conditions on inferred mechanical properties. Overall, this work provides a simplified but practical route for transforming deformation-based phenotypes into quantitative, loading-aware mechanical parameters for single-cell analysis.

## 1. Introduction

Microfluidic hydrodynamic deformation assays have become widely used platforms for high-throughput, single-cell, mechanical phenotyping. In hydrodynamic stretching systems, cells are deformed by extensional or shear stresses generated through microchannel geometries, and deformation is quantified from microscopy images via metrics such as projected area, contour shape, and deformation index [1,2]. In real-time deformability cytometry, cells traverse constriction or extensional regions under continuous flow, enabling label-free extraction of deformation descriptors at high throughput [3,4]. In inertial and pressure-driven microfluidic systems, deformation and trajectory-based features such as eccentricity or shape anisotropy are similarly used as mechanical proxies [5,6]. More recently, impedance-based and imaging-free approaches have further integrated electrical or optical signals with deformation-related metrics for label-free phenotyping [7,8,9,10,11].

Despite differences in device architecture and readout modality, these methods share a common analytical assumption: deformation-derived indices are treated as direct indicators of cellular mechanical properties. This assumption is useful for rapid population-level comparison but is not physically rigorous. Deformation is an emergent response determined jointly by cell size, hydrodynamic stress distribution, and intrinsic mechanical properties [12,13]. Consequently, direct comparison of deformation values across heterogeneous populations can introduce systematic bias, particularly when cell size distributions or local flow conditions differ.

In addition, living cells exhibit loading-dependent mechanical responses due to their complex and multi-component architecture. Specifically, their effective mechanical response arises from coupled contributions of the cortex, cytoskeleton, cytoplasm, and nucleus. At low to moderate stresses, cortical tension and cytoskeletal organization dominate deformation behavior [2,14,15,16], whereas higher stresses engage whole-cell deformation and nuclear mechanics [17,18,19,20]. Therefore, a single deformation descriptor is generally insufficient to capture the full mechanical response of heterogeneous cell populations across varying loading regimes.

Numerical modeling has been increasingly used to interpret deformation-based mechanical measurements in microfluidic assays. Early analytical models demonstrated that cellular deformation is determined not only by stiffness but also by cell size and confinement geometry and showed that hydrodynamic stress analysis combined with elastic theory can decouple these effects to extract mechanical properties [21]. Subsequent numerical studies extended this framework to larger deformation regimes by incorporating more complex cellular mechanical behaviors, including bulk elasticity, cortical elasticity, surface tension, and hyperelastic constitutive models, enabling more comprehensive interpretation of deformation responses [22]. However, existing numerical models have primarily addressed shear flow-based deformation assays. For microfluidic platforms involving impact-induced deformation regions, a general theoretical framework for directly converting deformation into mechanical properties remains unavailable. In this study, we therefore establish a simplified simulation mapping strategy to estimate apparent Young’s modulus (*E*) from experimentally measured diameter–deformation responses.

To address these limitations, we propose a simulation-based microfluidic workflow for estimating the apparent *E* of single cells from region-dependent deformation measurements. A three-region microfluidic channel is designed to generate spatially distinct hydrodynamic loading conditions, leading to region-specific deformation responses. Numerical simulations are used to construct calibration maps linking cell diameter, deformation, and *E* under each region’s flow condition. Based on these simulations, region-specific nonlinear surface-fitting models are developed to invert experimental diameter–deformation data into estimated *E* distributions.

We demonstrate the approach using primary T cells and K562 cells, showing that inferred *E* exhibits clear region dependence under different loading conditions. Overall, this work provides a practical bridge between image-derived deformation phenotypes and quantitatively interpretable, loading-aware single-cell mechanical parameters.

## 2. Materials and Methods

### 2.1. Numerical Simulation

**Figure 1:**
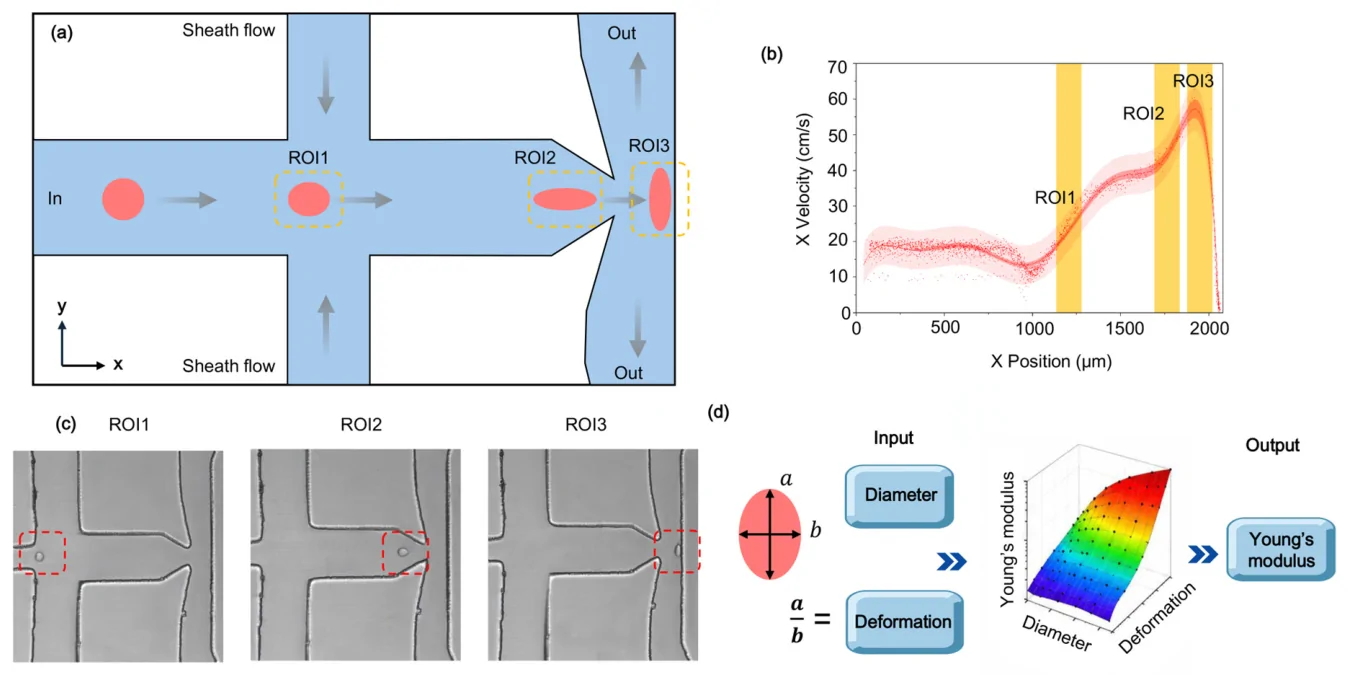
Working principle of the three-region simulation-based microfluidic deformation platform. (**a**) Schematic of the microfluidic chip design showing the three deformation regions (ROI1-ROI3). The red object represents a cell and arrows indicate the flow direction from the inlet and sheath-flow streams to the outlets. (**b**) Cell velocity distribution along the channel path, with ROI1-ROI3 indicated in orange. The shaded red region represents the 95% Confidence Band of x Velocity and 95% Prediction Band of x Velocity. (**c**) Representative experimental images of cell deformation. (**d**) Schematic of the deformation-to-*E* inversion workflow using cell diameter and deformation as inputs for simulation-based *E* estimation.

Numerical simulations were performed using ANSYS Fluent 2024 R1 (ANSYS Inc., Canonsburg, PA, USA) to establish the relationship among cell diameter, deformation, and E in the three regions of interest. The microfluidic channel was modeled as a three-dimensional geometry based on the chip design shown in Figure 1a, with a width of 80 μm and a height of 40 μm. A cell-sized elastic sphere was initially placed 40 μm downstream from the left inlet. The sphere diameter was varied from 5 to 30 μm. The fluid was assumed to be incompressible and Newtonian, with a density of 1005 kg∙$m^{-3}$ and a dynamic viscosity of 0.2 Pa∙s. The flow was treated as laminar, and gravity was neglected. The flow field was governed by the incompressible Navier–Stokes equations and solved using a pressure-based transient solver. Pressure inlet boundary conditions were applied, with 200 mbar at the sample-flow inlet and 180 mbar at the sheath-flow inlets. The outlet pressure was set as the reference pressure, and no-slip boundary conditions were applied to the channel walls. The moving sphere was treated as a solid linear elastic body. The assumption of a homogeneous, isotropic, linear, elastic cell is a widely adopted simplification in numerical studies of microfluidic deformability cytometry [21,22]. It effectively captures the primary correlation between cell size, hydrodynamic loading, and bulk deformation response and provides a computationally efficient basis for constructing the calibration mapping. This level of simplification is consistent with the goal of the present work to establish a low-cost and facile estimation framework. To generate the calibration dataset, different E values were assigned to the sphere while keeping Poisson’s ratio at 0.3. The E range covered 5000–24,000 Pa, with additional intermediate values used to improve the resolution of the calibration curves. Because the sphere underwent relatively large displacement in the channel, an overset mesh strategy was adopted. The main flow domain and the local flow region surrounding the sphere were meshed separately and assembled using an overset interface. The fluid domain was meshed using hexahedral elements, with local mesh refinement near the sphere. The total number of mesh elements was approximately 1.3 million. Six-degree-of-freedom motion was enabled for the moving sphere. The transient flow simulation was performed with a time step of 2 μs, and the total simulated time was 2.69 ms.

A one-way fluid-to-structure coupling strategy was used to calculate sphere deformation. Given that cell deformation in this system does not cause significant disturbance to the global flow field and the feedback of structural deformation to the flow field is relatively weak, the one-way coupling strategy can effectively balance calculation accuracy and computational efficiency. This simplification greatly reduces the computational cost of constructing the full calibration dataset, making the simulation-based mapping strategy practically feasible. The flow field was first solved, and the pressure distribution obtained from the transient flow simulation was then imported into the structural model as the external load. The sphere deformation was calculated using a linear, elastic, solid model under the imported pressure loading. Contact between the sphere and channel wall was included when wall collision occurred. The deformation of the sphere was quantified from the deformed geometry, and the resulting diameter–deformation data were used to construct the calibration dataset for *E* inversion. We acknowledge that the above simplifications will introduce certain deviations in the absolute values of the estimated apparent Young’s modulus. However, since the same set of assumptions is consistently applied to all calibration simulations and experimental inversion processes, the relative comparison between cell populations within the same platform remains internally consistent and reliable. Introducing two-way coupling and more realistic constitutive models (e.g., hyperelastic and cortical shell models) is an important direction for further improving model accuracy in future work.

### 2.2. Surface Fitting and Estimated Young’s Modulus Inversion

To convert simulated deformation responses into a quantitative E estimation framework, region-specific empirical surface-fitting models were constructed based on the simulated dataset, which consisted of sphere diameter (d), deformation parameter ($D_{def}$), and assigned Young’s modulus (*E*). Because the deformation–diameter–*E* relationship exhibited distinct nonlinear characteristics across different flow regimes, ROI1, ROI2, and ROI3 were modeled separately to match their respective hydrodynamic loading conditions and deformation mechanisms.

The rationale for adopting region-specific models rather than a single unified regression model is explained as follows. First, there are essential differences in flow fields and deformation mechanisms among the three regions. ROI1 is dominated by weak shear flow with small cell deformation, ROI2 is a mixed shear–extensional transition zone with intermediate deformation, and ROI3 is governed by impact extensional flow with large deformation and pronounced saturation effects. The mathematical forms of the diameter–deformation–modulus relationship differ substantially across the three regions, so a unified regression model cannot simultaneously capture three distinct nonlinear patterns, which would significantly reduce the overall fitting accuracy. Second, measurement events in the three ROIs are independent of each other. The three ROIs are spatially separated detection positions, and experimental images capture independent cell events at different locations, rather than continuous tracking of the same cell. Therefore, there is no physical requirement for modulus to transition continuously across regions, and region-specific modeling does not cause discontinuity problems in practical applications. Third, partitioned modeling enables the optimal model form for each region. We can select the most suitable model structure for each deformation regime (exponential model for small deformation, rational polynomial for large deformation), maximizing the fitting and prediction accuracy of each individual region.

Regarding potential inter-region discontinuity, since each cell event is assigned to only one ROI based on its spatial location and processed by the corresponding calibration model alone, there is no scenario where a single data point spans two regions and encounters a discontinuous jump in predicted *E*. All calibration surfaces are defined strictly within their respective spatial and parameter ranges, and no artificial discontinuity is introduced into the actual data processing workflow.

For ROI1 and ROI2, the deformation fell within the small-to-intermediate deformation regime. Specifically, ROI1 is located upstream of the constriction, where the flow field remains stable and is dominated by weak shear stress. In this small-deformation region, the mechanical response can be reasonably approximated by smooth exponential-type scaling laws, and a baseline-shifted exponential model was used:

(1) $$E=10^{2}\left(23.56e^{-20.02\left(D_{def}-0.0061d-1\right)}+48.32\right)$$

ROI2 sits in the geometric transition zone between the tapered channel and the impact region, where shear flow, extensional flow, and local flow redistribution effects coexist. The spatially non-uniform hydrodynamic stress distribution leads to stronger coupling among cell diameter, deformation, and Young’s modulus, which complicates the three-dimensional mapping relationship. To account for this enhanced diameter–deformation coupling, a two-variable coupled exponential interaction model was adopted for ROI2:

(2) $$E=5523.5+5,871,300e^{\left(-0.0383d-6.4221D_{def}+0.1454dD_{def}\right)}$$

In contrast, ROI3 corresponds to the impact/stagnation region with well-defined extensional flow and pressure distribution, where cells enter the large-deformation regime and nonlinear geometric effects as well as pronounced deformation saturation become dominant. In this region, deformation is incorporated into the model in a reciprocal form, reflecting its inverse relationship with apparent stiffness. Therefore, a rational polynomial model was used:

(3) $$E=\frac{\left(2053.17+109.37d-0.97d^{2}\right)}{\left(D_{def}-0.95\right)}-\left(-1943.86-136.91d+1.95d^{2}\right)$$

During implementation, lower-bound constraints were imposed on both the diameter term and the denominator term to prevent non-physical predictions and numerical instability.

The fitted models served as region-specific calibration surfaces for *E* inversion. The overall predictive performance of the models was strong, as indicated by the coefficients of determination (*R*^2^): *R*^2^ = 0.91 for ROI1, *R*^2^ = 0.85 for ROI2, and *R*^2^ = 0.99 for ROI3. The relatively lower *R*^2^ in ROI2 stems from two main physical causes. First, as the transitional interval between small and large deformation, ROI2 features progressively emerging geometric nonlinearity and the strongest diameter–deformation coupling. A compact empirical formula cannot fully capture all nonlinear details across the full ranges of diameter and modulus, resulting in relatively larger fitting residuals. Second, the complex superposition of multiple flow patterns in this transition zone increases the complexity of the deformation–mechanics relationship, raising the difficulty of empirical surface fitting. We also attempted higher-order polynomials and more sophisticated nonlinear model forms for ROI2, but these structures introduced notable overfitting risks at the boundaries of the calibration range and impaired generalization performance for experimental data. Accordingly, the current bivariate, coupled, exponential model was selected to strike a balance among fitting accuracy, model simplicity, and generalization capability.

We further quantitatively evaluated the influence of this fitting uncertainty on the accuracy and reliability of apparent Young’s modulus estimation. Analysis of the calibration dataset shows that the fitting residuals in ROI2 are mainly distributed within a relative range of ±15% with respect to the assigned *E* values, which is acceptable for comparative mechanophenotyping on the same platform. Importantly, the fitting error exhibits no systematic bias with respect to cell diameter or stiffness level and presents as random scatter rather than a systematic offset. Therefore, it does not distort the overall stiffness ranking or the relative magnitude of mechanical differences between cell populations, and the statistical conclusions of inter-population comparisons remain robust. At the single-cell level, the measurement uncertainty in ROI2 is higher than that in ROI1 and ROI3; however, for population-level statistical analysis with sufficient sample size (thousands of cells per group in this study), the random fitting error is effectively averaged out, ensuring the reliability of apparent population-level *E* results.

For experimental data processing, each cell event was first assigned to the corresponding ROI based on its spatial location, after which the measured diameter and deformation values were input into the corresponding calibration model to compute the apparent Young’s modulus automatically. All models were applied strictly within their calibrated parameter ranges, and out-of-range events were excluded or flagged to ensure physical consistency.

### 2.3. Software Implementation

A custom Python-based desktop application named Cell Mechanical Mapping System was developed to automate *E* estimation and visualization. The graphical user interface was constructed using Tkinter, while Pandas was used for data import, preprocessing, and batch calculation. Matplotlib was used for three-dimensional surface visualization, two-dimensional *E* mapping, and figure export.

The software accepts experimental data files containing the measured cell diameter and deformation parameter. After importing the experimental dataset, the user selects the corresponding ROI-specific fitting model. The software then calculates the estimated *E* for each cell event using the fitted calibration equation.

In addition to batch *E* estimation, the software provides several visualization and export functions. First, the estimated *E* values can be displayed in a three-dimensional mapping view, where the axes represent cell diameter, deformation, and *E*. Second, equal- Young’s modulus curves can be generated by inversely solving the fitted surface equation at selected *E* levels. Third, a two-dimensional filled contour map can be rendered to show the *E* distribution in the diameter–deformation space, with experimental cell data overlaid as scatter points. The calculated *E* table and generated figures can be exported for further statistical analysis and manuscript preparation.

### 2.4. Cell Culture and Sample Preparation

K562 cells, an established human leukemia cell line, were obtained from the China Center for Type Culture Collection (CCTCC; catalog no. GDC0094), and the use of this cell line did not involve the collection of additional human specimens. The cells were maintained in RPMI 1640 medium(Gibco, Grand Island, NY, USA) supplemented with 10% fetal bovine serum (FBS) and 1% penicillin–streptomycin. Human primary T cells used in this study were isolated from peripheral blood collected from healthy donors. Peripheral blood mononuclear cells (PBMCs) were first isolated from whole blood samples, and primary T cells were subsequently purified via CD3/CD28 magnetic bead-based enrichment. The sorted T cells were cultured in X-VIVO medium supplemented with 2% FBS and cytokines (IL-2, IL-7, and IL-15). Both cell types were recovered from frozen stocks by rapid thawing at 37 °C and cultured in a humidified incubator at 37 °C with 5% CO_2_. Cells were maintained as suspension cultures and routinely adjusted to a density of 0.3–0.5 × 10^6^ cells/mL by medium replacement when required.

### 2.5. Experimental Workflow

All single-cell mechanical phenotyping experiments were independently repeated at least three times, and datasets from all replicates were merged for subsequent statistical analysis to ensure result reproducibility and statistical reliability. For single-cell mechanical phenotyping, the cell suspension and sheath flow were introduced into the microfluidic chip using a pressure pump (FluidicLab, Shanghai, China), with viscoelastic flow details as in our previous work [22]. Three regions of interest (ROIs) with distinct hydrodynamic loading profiles were predefined along the microchannel flow path: ROI1 corresponds to the upstream low-shear region under mild extensional flow, ROI2 corresponds to the tapering transition region with progressively elevated flow velocity and shear stress, and ROI3 corresponds to the impact stagnation region subjected to the strongest hydrodynamic loading. Single cells were hydrodynamically focused and passed through the deformation region sequentially, where cell images were recorded by a high-speed camera (Mshot, Guangzhou, China) (1000 frames/s). For image processing, raw, high-speed micrographs were first preprocessed with background subtraction and adaptive grayscale thresholding. For each valid single-cell event, the cell contour was automatically extracted from the image sequences using Droplet Morphometry and Velocimetry (DMV) software, which was developed using MATLAB R2025a (MathWorks, Inc., Natick, MA, USA) [23]. The equivalent cell diameter was calculated as the geometric mean of the major and minor axes of the fitted elliptical contour, and the deformation parameter was defined as the ratio of the major axis length to the minor axis length. For each single-cell event, the cell contour was extracted from the recorded images using Droplet Morphometry and Velocimetry (DMV) software [23]. The measured diameter–deformation pairs were then imported into the simulation-calibrated fitting model to estimate the apparent Young’s modulus of individual cells for downstream statistical analysis. The total number of analyzed single-cell events for each group is as follows: 607 K562 cells and 627 primary T cells in ROI1; 598 K562 cells and 598 primary T cells in ROI2; 627 K562 cells and 481 primary T cells in ROI3.

## 3. Results

### 3.1. Three-Region Microfluidic Deformation Platform

Figure 1a illustrates the working principle of the simulation-based microfluidic deformation method. The channel was designed to generate three characteristic deformation regions of interest (ROIs), denoted as ROI1 (upstream low-shear region), ROI2 (tapered transition region), and ROI3 (impact stagnation region), where cells experience distinct hydrodynamic loading conditions. Cells were introduced into the main channel and transported downstream by fluid flow. With the combined effects of sheath-flow focusing and gradual channel geometry variation, cells experienced progressively increased hydrodynamic stresses and deformation along the flow path from ROI1 to ROI3 (Figure 1b). Similar multi-region deformation designs have been previously demonstrated by our group for obtaining multi-stress-level mechanical phenotypes, where cells undergo sequentially different deformation states under controlled flow conditions [24].

The enlarged views of ROI1-ROI3 show the local channel structures and representative experimental images of cells in each region (Figure 1c). Cell morphology changed progressively during transport, indicating that the three regions provided different deformation states rather than repeated measurements under the same condition. For each cell event, the cell contour was extracted from microscopy images, and the cell diameter and deformation parameter were calculated from the major (a) and minor (b) axes of the cell profile (Deformation = $\frac{a}{b}$).

The overall workflow is shown in Figure 1d. Experimentally measurable parameters, including cell diameter and deformation, were used as inputs. A numerical simulation model then established the relationship among cell size, deformation, and *E*. Based on the simulated calibration dataset, a three-dimensional fitted surface was constructed for each ROI. The *E* of individual cells could then be inversely estimated from their measured diameter and deformation. 

### 3.2. Simulation-Based Deformation–E Mapping and Apparent Young’s Modulus Estimation

**Figure 2:**
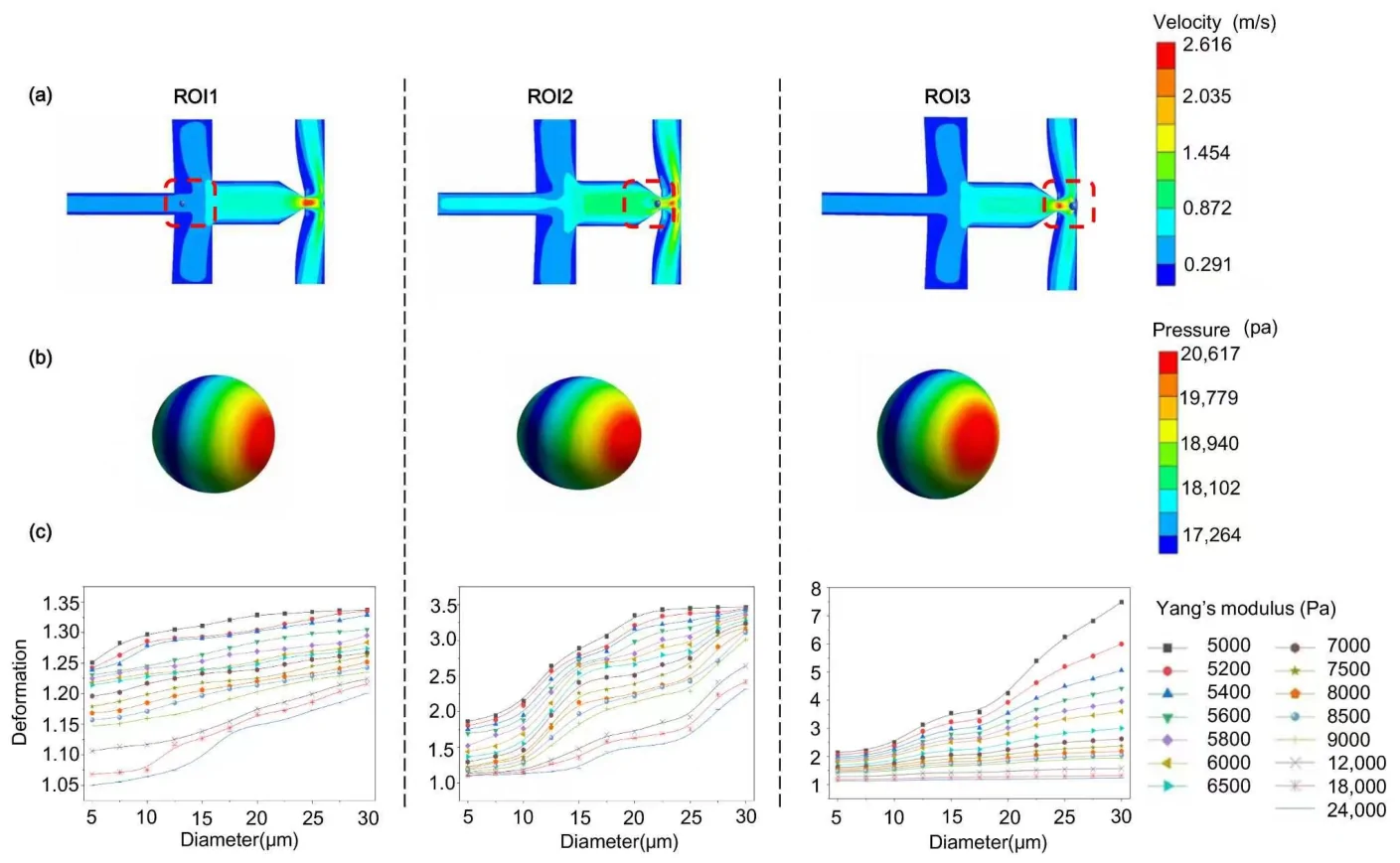
Numerical simulation of region-specific hydrodynamic loading and size-dependent deformation–*E* relationships. (**a**) Simulated flow fields in ROI1, ROI2, and ROI3; the color map indicates local flow velocity as the cell-sized sphere passes through each region. (**b**) Simulated pressure distributions acting on the sphere in the three ROIs. (**c**) Equal-Young’s-modulus curves obtained from simulations with different sphere diameters and assigned E.

To establish the deformation–*E* relationship under different loading conditions, three-dimensional numerical simulations were performed for the three regions of interest. As shown in Figure 2a, the velocity field varied markedly among ROI1, ROI2, and ROI3. Local acceleration and flow redistribution near the channel junction generated distinct hydrodynamic environments in the three regions.

Figure 2b shows the simulated pressure distribution acting on the cell-sized elastic sphere in the three ROIs and the corresponding deformation responses. The pressure loading varied among ROI1–ROI3, as reflected by the progressively increased deformation from ROI1 to ROI3. These results confirm that the three regions provide distinct hydrodynamic loading conditions. Such multi-level mechanical stimulation is important because different stress regimes may reveal different aspects of cellular mechanical responses [2,4,24].

Subsequently, cell-sized elastic spheres with different diameters and *E* values were simulated to establish the deformation–mechanics relationship. The sphere diameter ranged from 5 to 30 μm, and *E* was varied from 5000 to 24,000 Pa. For each combination of diameter and *E*, the deformation response was calculated under the corresponding simulated pressure loading. The resulting deformation profiles were represented as equal-*E* curves, as shown in Figure 2c.

The simulation results demonstrate that deformation is a coupled response determined by both cell size and *E* under hydrodynamic loading. In general, deformation increased with increasing cell diameter but decreased with increasing *E*. In ROI1, cells experienced relatively low external forces, resulting in limited deformation and a relatively gradual variation across different diameters and *E* values. In ROI2, the increased hydrodynamic loading led to larger deformation and a more pronounced dependence on mechanical properties. In ROI3, where cells underwent strong deformation, the deformation response was substantially amplified across the investigated size range, indicating enhanced sensitivity to variations in cellular mechanical properties.

**Figure 3:**
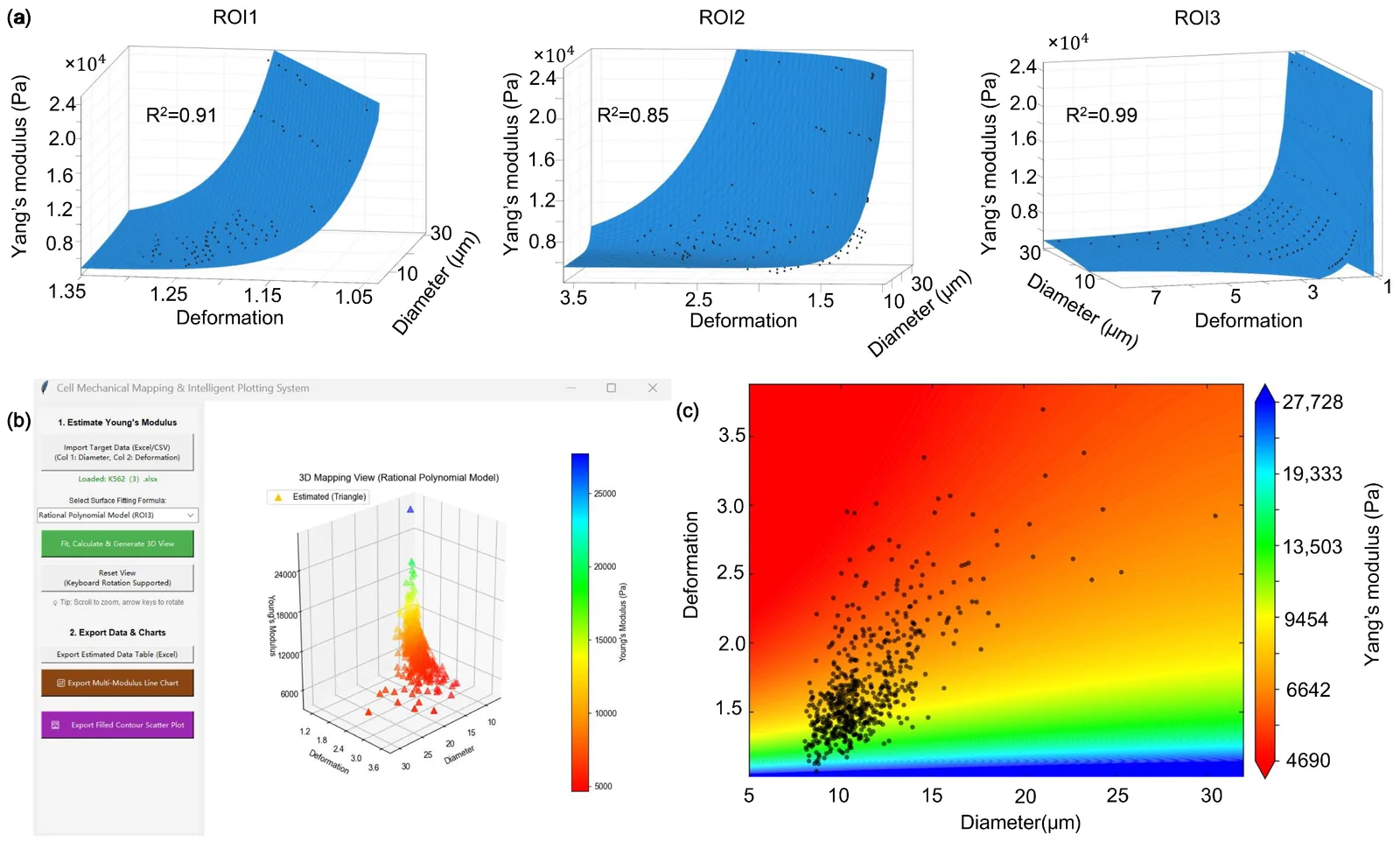
Surface fitting and software-based automatic calculation of apparent Young’s modulus. (**a**) Region-specific fitted surfaces for ROI1, ROI2, and ROI3, with corresponding R^2^ values. (**b**) Interface of the custom Cell Mechanical Mapping System used for importing diameter–deformation data and calculating apparent Young’s modulus. (**c**) Software-exported *E* distribution map. Black points represent experimentally measured cell diameter–deformation pairs, while the background color map represents the calculated Young’s modulus distribution.

Based on the simulated dataset, region-specific nonlinear surface models were constructed for ROI1, ROI2, and ROI3. As shown in Figure 3a, the fitted surfaces describe the relationship among cell diameter, deformation, and assigned *E* under different hydrodynamic loading conditions. The formulations of these models are provided in Section 2, and the fitting performance was evaluated using the coefficient of determination (R^2^), with values of 0.91, 0.85, and 0.99 for ROI1, ROI2, and ROI3, respectively. These results indicate that the simulated diameter–deformation–*E* relationships can be approximated using empirical surface-fitting models. To address the limitation of relying solely on R^2^ for model assessment, we used cross-validation analysis to verify model robustness. Cross-validation errors closely matched training errors, confirming stable generalization performance and the absence of overfitting across all three models. Leave-one-sample-out tests further demonstrated that eliminating individual calibration data points would not induce significant variation in prediction accuracy.

The fitted equations were then integrated into a custom software interface for automated apparent Young’s modulus estimation. As shown in Figure 3b, users can import experimental diameter–deformation data and select the corresponding ROI-specific model. The software calculates the *E* value for each cell event and provides visualization outputs. Figure 3c shows an example of the *E* mapping result, where experimental data points are overlaid on the fitted *E* distribution in the diameter–deformation space. This visualization allows the mechanical position of each cell event to be interpreted relative to the simulated deformation–*E* mapping surface.

### 3.3. Experimental Deformation Analysis and Region-Dependent Apparent Young’s Modulus Estimation

After establishing the simulation-based *E* estimation model, the method was applied to experimental measurements of primary T cells and K562 cells. Primary T cells and K562 cells were selected as representative immune and hematological cancer models, respectively [25,26,27]. Both cell types are widely used in studies of immune function, cancer biology, and cellular biomechanics, making them suitable models for evaluating single-cell mechanical phenotyping.

**Figure 4:**
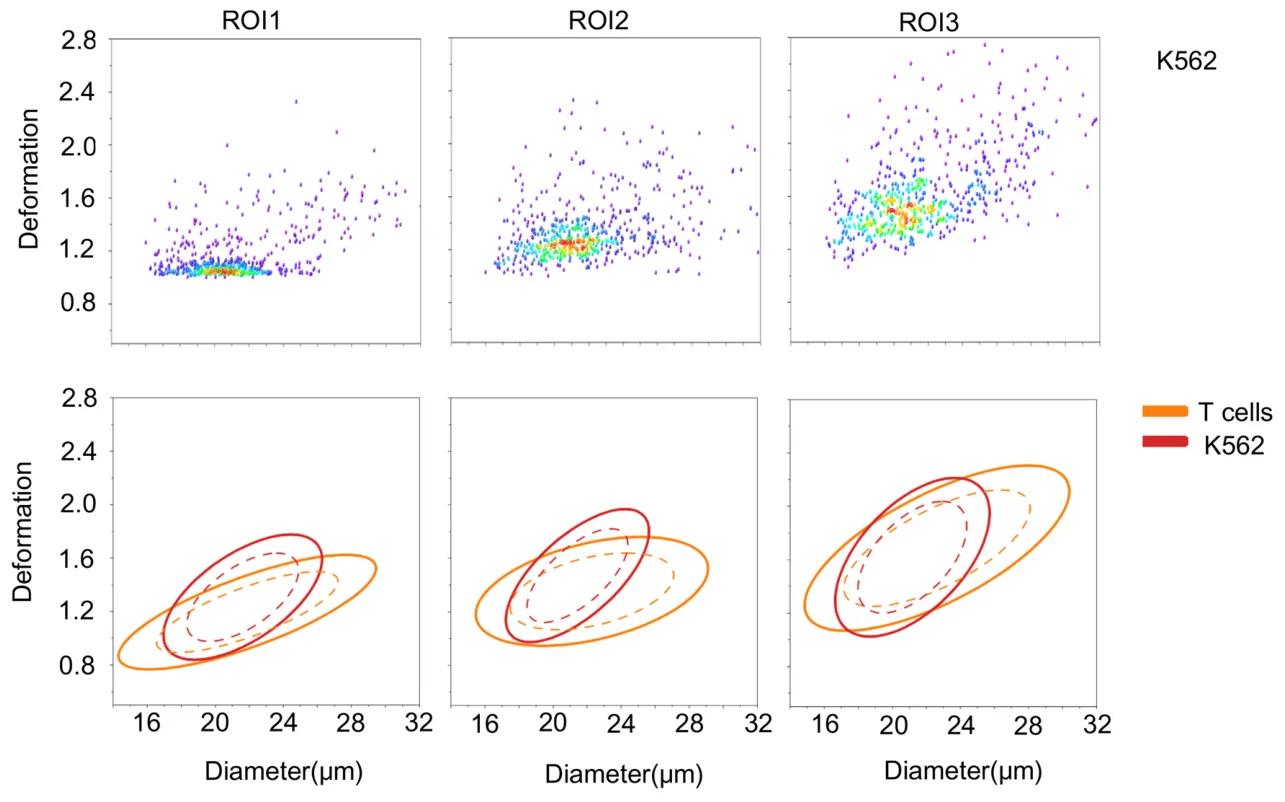
Experimental deformation measurement of primary T cells and K562 cells in ROI1-ROI3. Density maps of K562 cell diameter–deformation distributions in the three regions of interest. Contour plots comparing primary T cells and K562 cells in diameter–deformation space. Orange contours represent primary T cells and red contours represent K562 cells; solid ellipses indicate 70% confidence regions and dashed ellipses indicate 45% confidence regions.

Figure 4 shows the measured diameter–deformation density map of K562 cells as an example. Subsequently, contour plots were generated to compare the diameter–deformation distributions of primary T cells and K562 cells under the same deformation regions. The two cell populations exhibited distinct distribution patterns, while considerable overlap remained. As expected, the overall deformation increased progressively from ROI1 to ROI3 due to the increasing hydrodynamic loading. Notably, the difference between the two cell populations became less pronounced in ROI3, where both cell types experienced larger deformation.

Although the diameter–deformation distributions revealed differences between primary T cells and K562 cells, these deformation-based representations alone were insufficient to determine which population possessed higher intrinsic mechanical stiffness. This limitation highlights that deformation is an indirect mechanical readout influenced by multiple factors, including cell size, external loading, and cellular mechanical response. Therefore, further conversion from deformation measurements to apparent Young’s modulus is required for a more quantitative comparison of cellular mechanical properties.

Independent-sample Student’s *t*-tests were performed to assess the statistical significance of intergroup mechanical differences, with Welch’s correction applied when unequal variance was confirmed by Levene’s test. K562 cells exhibited significantly higher apparent Young’s modulus than primary T cells in both ROI1 (median: 103.3 vs. 50.0 kPa; *p* < 0.001) and ROI2 (median: 47.7 vs. 32.3 kPa; *p* < 0.001). In contrast, primary T cells showed a significantly higher apparent Young’s modulus than K562 cells in ROI3 (median: 12.3 vs. 10.8 kPa; *p* < 0.001). All intergroup differences reached high statistical significance, confirming that the observed region-dependent mechanical phenotypes are robust and biologically distinguishable.

**Figure 5:**
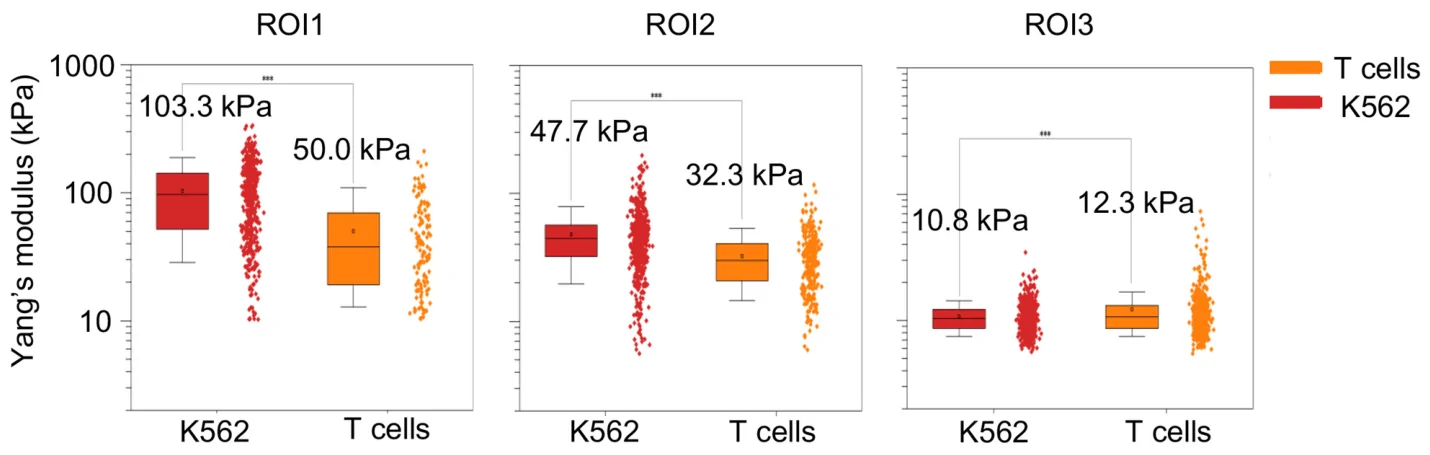
Region-dependent apparent Young’s modulus estimation for primary T cells and K562 cells. E values were calculated from experimentally measured cell diameter and deformation using the ROI-specific fitted calibration surfaces. The distributions compare the estimated E of the two cell populations in ROI1, ROI2, and ROI3, revealing loading-dependent differences between primary T cells and K562 cells. Data are mean ± SD. Statistical significance was determined by Student’s *t*-test; *** *p* < 0.001.

Using the ROI-specific surface models, the apparent Young’s modulus of individual primary T cells and K562 cells was estimated from their measured diameter and deformation values. Figure 5 summarizes the *E* distributions obtained from ROI1, ROI2, and ROI3. Compared with the diameter–deformation analysis, the converted *E* values provided a clearer mechanical comparison between the two cell populations.

The estimated *E* showed clear, region-dependent differences between the two cell populations. In ROI1 and ROI2, K562 cells exhibited higher *E* than primary T cells, with median values of 103.3 vs. 50.0 kPa in ROI1 and 47.7 vs. 32.3 kPa in ROI2, respectively. The difference in *E* was most pronounced in ROI1. In contrast, this trend was reversed in ROI3, where primary T cells showed a slightly higher *E* than K562 cells (12.3 vs. 10.8 kPa). These results indicate that the inferred mechanical contrast between cell populations depends on the local external loading conditions.

## 4. Discussion

Microfluidic deformation assays provide a powerful approach for high-throughput, label-free, single-cell mechanophenotyping. However, deformation itself is not a direct mechanical parameter, as it is jointly determined by cell size, local hydrodynamic loading, and cellular mechanical properties. This limitation was also observed in the present study, where primary T cells and K562 cells showed partially different diameter–deformation distributions. However, these distributions did not provide a clear mechanical interpretation, as it remained difficult to determine which population was mechanically stiffer. Therefore, converting deformation measurements into a more interpretable mechanical parameter is important for quantitative mechanophenotyping.

Young’s modulus (*E*) provides a commonly used descriptor for cellular stiffness, but deformation-to-*E* conversion remains challenging for microfluidic systems with complex loading conditions. While theoretical and numerical models have been established for shear- or extensional-flow-based deformability cytometry, a general model for impact-induced or multi-region deformation systems remains unavailable. Here, we developed a simplified simulation-based estimation strategy, where numerical simulations established diameter–deformation–*E* relationships under different loading conditions. The resulting apparent *E* values should be interpreted as loading-specific mechanical parameters rather than absolute intrinsic material constants.

From a practical standpoint, this simulation-calibrated estimation method provides a low-cost and facile approach for rough evaluation of cellular apparent stiffness, with distinct advantages over traditional cell mechanical characterization techniques. Conventional precision methods, including atomic force microscopy, micropipette aspiration, and optical tweezers, generally rely on dedicated, high-cost instrumentation, require specialized operational expertise, and suffer from limited throughput, which restrict their broad application in routine biological laboratories. By contrast, the proposed framework is built upon standard microfluidic deformation measurements, cell diameter and deformation values that are already routinely acquired in deformability cytometry experiments. Once the simulation-derived calibration surfaces are pre-constructed for a given channel design, apparent *E* values can be automatically computed in a high-throughput batch manner using the fitted empirical models, with no need for additional experimental hardware or advanced computational background. This high accessibility enables ordinary laboratories to perform quantitative single-cell mechanophenotyping using conventional microfluidic platforms and offers a practical tool for preliminary mechanical screening and population-level comparative studies.

To independently validate the reliability and physical rationality of the apparent Young’s modulus inverted by our simulation-fitting framework, we systematically compared our estimated stiffness ranges with benchmark mechanical characterization results of primary T cells and K562 cells reported in the peer-reviewed literature, covering three gold-standard experimental techniques: atomic force microscopy (AFM), micropipette aspiration, and real-time deformability cytometry (RT-DC) [4,19,27]. The apparent E values of primary T cells and K562 cells derived across ROI1–ROI3 span 10.8–103.3 kPa, which falls within the same order of magnitude as previously published stiffness data for the two cell lines. For suspended K562 leukemia cells, AFM indentation measurements record elastic moduli ranging from hundreds of Pascals to ~120 kPa under quasi-static contact loading [26]; micropipette aspiration captures the stiffness of hematopoietic cells at the tens-to-hundreds-of-Pascals scale under gentle suction [27]. For human primary T lymphocytes, AFM single-cell probing and microplate stretching report apparent stiffness distributed between ~100 Pa and 60 kPa [25], while RT-DC hydrodynamic stretching yields cell elastic parameters within the kPa regime under continuous flow-induced deformation [3,5]. The consistent magnitude between our simulation-calibrated outputs and literature benchmarks verifies that the proposed inversion framework generates physically meaningful cellular stiffness readouts.

Notably, absolute numerical discrepancies between our E results and literature data are a universal challenge in cellular biomechanics, originating from fundamental differences in loading mode, deformation strain rate, maximum deformation amplitude, cell confinement state, and constitutive model assumptions across distinct measurement platforms [4,13]. The modulus extracted herein is explicitly defined as loading-condition-dependent apparent stiffness, rather than the intrinsic material elastic constant of living cells. To achieve stricter direct experimental independent validation beyond literature cross-comparison, our follow-up research will conduct parallel AFM characterization on identical batches of primary T cells and K562 cells, enabling one-to-one paired comparison between our microfluidic multi-region estimation and a well-established contact-based mechanical measurement technique.

Interestingly, the difference in *E* between the two cell populations decreased from ROI1 to ROI3, with ROI1 exhibiting the most pronounced mechanical contrast. This observation is consistent with our previous finding that deformation-based mechanical differences can be more apparent under relatively low external loading conditions [28]. One plausible explanation is that the mechanical response under these loading conditions is more strongly influenced by cortical and cytoskeletal properties, where differences in cytoskeletal organization may contribute substantially to the observed deformation response [14,15,16]. It should be emphasized that this mechanistic interpretation is drawn from published evidence and has not been directly verified in the present study.

In contrast, primary T cells exhibited slightly higher *E* than K562 cells in ROI3, where cells experienced stronger deformation. This difference may be associated with the relatively large nuclear volume fraction of T cells. Because T cells typically possess a relatively high nucleus-to-cytoplasm ratio, nuclear mechanics may contribute more substantially to the whole-cell mechanical response under large-deformation conditions [17,18,19,20,29]. The nucleus is mechanically distinct from the surrounding cytoplasm and can act as a stiff inclusion during confined or compressive deformation [17,18,19,20]. Nevertheless, this remains a literature-based inference, and further dedicated experiments are needed to confirm the underlying structural mechanism in future work.

Therefore, stronger hydrodynamic loading in ROI3 may engage nuclear mechanical resistance more effectively than the lower-deformation conditions in ROI1 and ROI2.

From a methodological perspective, the core analytical logic of “numerical simulation calibration + empirical surface inversion” can be referenced and adapted by various microfluidic deformation platforms, but the specific calibration surfaces and modulus values are not universally applicable across devices. For different platforms such as shear-flow cell stretching systems and constriction-based deformability cytometry, researchers can follow the same analytical workflow: reconstruct the corresponding channel geometry in numerical simulations, set boundary conditions matching actual experimental parameters, and build a platform-specific calibration surface linking cell diameter, deformation, and apparent Young’s modulus to convert deformation phenotypes into quantitative mechanical parameters.

It should be emphasized that the estimated apparent Young’s modulus is a loading-dependent phenotypic parameter rather than an absolute intrinsic material constant. ROI1, ROI2, and ROI3 reflect cellular mechanical responses under low, moderate, and high hydrodynamic loading, respectively, and their results complement each other to collectively form a multi-scale mechanical profile of cells. Due to differences in loading modes, stress distributions, and deformation regimes, absolute *E* values obtained from different microfluidic platforms cannot be directly interchanged as universal material constants. Nevertheless, this simulation-calibrated analytical framework provides a unified quantitative analysis paradigm, laying a methodological foundation for advancing the cross-platform standardization of single-cell mechanophenotyping.

At present, numerous microfluidic deformability methods have been developed, but the reported deformation indices are often device-specific and difficult to compare across platforms. Even Young’s modulus values may vary substantially depending on the measurement technique, loading geometry, deformation regime, constitutive model, and data-processing strategy. This lack of standardization limits direct comparison between different studies. Therefore, the absolute *E* values obtained in this study should not be interpreted as universal Young’s modulus values. Instead, they provide a simulation-informed and internally consistent basis for comparing different cell populations within the same platform. By transforming deformation measurements into region-dependent *E* values, this approach may contribute to future standardization of microfluidic single-cell mechanophenotyping.

Looking ahead, several optimization directions can be pursued to further advance this strategy and promote the standardization of single-cell mechanophenotyping. First, the current homogeneous, linear, elastic cell model can be upgraded to more biologically realistic constitutive frameworks, such as viscoelastic models and multi-layered structural models that explicitly decouple the mechanical contributions of the cell cortex, cytoplasm, and nucleus. These improvements will allow more accurate characterization of rate-dependent mechanical behaviors and load-dependent structural responses, extending the method from single apparent modulus estimation to multi-parameter comprehensive mechanical phenotyping. Second, the current device-specific calibration workflow can be generalized into a unified simulation pipeline adaptable to various microfluidic geometries and loading modes (e.g., shear flow, constriction extrusion, impact deformation). Such a generalized framework would help eliminate platform-dependent biases and enable cross-platform comparability of mechanical parameters. Third, the integration of machine learning algorithms with numerical simulation can further improve the accuracy and efficiency of modulus inversion, enabling real-time mechanical parameter estimation directly from raw cell images. With continuous model refinement and multi-laboratory validation across diverse cell types and application scenarios, this simulation-based mapping approach is expected to facilitate the establishment of standardized quantitative protocols for microfluidic single-cell mechanophenotyping.

## Data Availability

The raw data supporting the conclusions of this article will be made available by the authors on request.
